# A high-resolution assessment of climate change impact on water footprints of cereal production in India

**DOI:** 10.1038/s41598-021-88223-6

**Published:** 2021-04-22

**Authors:** Santosh S. Mali, Paresh B. Shirsath, Adlul Islam

**Affiliations:** 1 ICAR-Research Complex for Eastern Region, Farming System Research Centre for Hill and Plateau Region, Ranchi, 834 010 India; 2 CGIAR Research Program on Climate Change, Agriculture and Food Security (CCAFS), Borlaug Institute for South Asia (BISA), International Maize and Wheat Improvement Centre (CIMMYT), New Delhi, 110012 India; 3 Natural Resource Management Division, Indian Council of Agricultural Research (ICAR), New Delhi, 110012 India

**Keywords:** Climate-change impacts, Hydrology

## Abstract

Water footprint (WF), a comprehensive indicator of water resources appropriation, has evolved as an efficient tool to improve the management and sustainability of water resources. This study quantifies the blue and green WF of major cereals crops in India using high resolution soil and climatic datasets. A comprehensive modelling framework, consisting of Evapotranspiration based Irrigation Requirement (ETIR) tool, was developed for WF assessment. For assessing climate change impact on WF, multi-model ensemble climate change scenarios were generated using the hybrid-delta ensemble method for RCP4.5 and RCP6.0 and future period of 2030s and 2050s. The total WF of the cereal crops are projected to change in the range of − 3.2 to 6.3% under different RCPs in future periods. Although, the national level green and blue WF is projected to change marginally, distinct trends were observed for Kharif (rainy season—June to September) and rabi (winter season—October to February) crops. The blue WF of paddy is likely to decrease by 9.6%, while for wheat it may increase by 4.4% under RCP4.5 during 2050s. The green WF of rabi crops viz. wheat and maize is likely to increase in the range of 20.0 to 24.1% and 9.9 to 16.2%, respectively. This study provides insights into the influences of climate change on future water footprints of crop production and puts forth regional strategies for future water resource management. In view of future variability in the WFs, a water footprint-based optimization for relocation of crop cultivation areas with the aim of minimising the blue water use would be possible management alternative.

## Introduction

Achieving zero hunger remained one of the primary development goal, and over the years India has achieved phenomenal growth in food production^1^ to feed its increasing population. The food grain production in India has increased from 82 million tonnes in 1960s to record 285 million tonnes during 2018–2019, the population during this time increased by three folds^2^. Food production is dependent upon the blue and green water resources^3^, and its spatio-temporal distribution is affected by climate change and variability^4–6^. The assured supply of green water is important for food security of India as 51.7% (72.2 million ha) of the net sown areas (139.5 million ha) is still rainfed^2^, and even on irrigated crop lands, green water has significant contribution in agricultural production^7^. Excessive withdrawals of non-renewable blue water resources to compensate green water deficit in longer run might lead to resource degradation; leading to increased vulnerability of the entire agricultural production systems especially under the threats posed by increased climatic variability and change.

The increased climatic variability, depleting glaciers, heat waves, rising sea level, frequent floods and droughts are impacting agriculture in different ways. Recent estimates using bias corrected projections indicate a warmer (3–5 °C) and wetter (13–30%) climate in South Asia in the twenty-first century^8^. Climate change has both direct and indirect impacts on ecosystems, social economics, agriculture and is likely to intensify the pressure on global water resources^9^. Changes in the availability of water, particularly for agriculture, due to climate change have been observed and reported globally^10^. Many climate adaptation and mitigation strategies with respect to water management in agricultural sector are being practiced at various levels (e.g., farm, irrigation scheme, watershed/aquifer, river basin, and national levels). Apart from social and policy level measures, the water sector particularly considers promoting efficient on-farm water management (water-saving devices, improving efficiency of water distribution and use, reusing waste water)^11^, managed aquifer recharge^12^, management of artificial and natural reservoirs^13, 14^ and geographical shifting of cultivation areas of some crops^15^ as potential alternatives for adapting and mitigating the impacts of climate change on water resources. Crop management practices like early planting and use of cultivars most suitable for warmer climates have also been practiced in some regions of the world^16, 17^.

Water footprint (WF) concept^18^ is regarded as a comprehensive indicator of water resources appropriation^19^. It consists of three components, viz. green, blue, and grey and is defined as the total volume of water used to produce the commodity. Since the introduction of WF concept, several studies reported WFs assessment both globally and regionally^20–23^. These analysis focussed on WF of commodities at different spatial scales, either hydrological unit^24–29^ or administrative units^30–35^. The commodity-wise assessment are also available for majority of agricultural products like milk^36^, farm animal products^37^, food grains^32^, either globally^38^ or regionally^22, 39^. Understanding the climate linkages with future water footprints is vital to formulate the advanced mitigation and adaptation mechanisms. In agricultural systems, climate change and its variability have profound impact on WFs^3, 4^, therefore, modelling the spatial and temporal variability of the climate change impacts will effectively alleviate the uncertainties which WF could be suffered from^40^.

The WF is inextricably interrelated to the climatic features and previous research works have shown that climate change had a considerable impact on WF of crop production^41–44^. The influence of climate change on regional irrigation water demand varies greatly among different geographical regions^45, 46^. Elbeltagi et al.^47^ reported that the crop evapotranspiration (ET_c_) of maize and wheat is projected to increase over western Nile delta while the green WF was predicted to decrease in the western and eastern parts of the Nile delta. Their study concluded that the eastern delta will be the optimal location for saving blue water accounts. Konar et al.^48^ used the H08 global hydrologic model and stated that trade liberalization leads to greater virtual water trade, making it a potentially important adaptation measure to continuously changing climate. The climate change impact analysis was helpful in comparative analysis of sensitivity of WFs to regional climate changes^42^. Therefore, research on the effects of climate change on the blue and green WFs of crop production is of great significance for guiding agricultural management to cope with climate change.

Over the years, WF has evolved as a new tool to assess the consumption and water use. The WF is a spatially and temporally explicit indicator that looks at both direct and indirect water use by the consumer or producer. It also provides vital data and scientific basis for making informed decisions on use of limited water resources sustainably. Although several authors reported studies on WF in agriculture for India^22, 27, 32, 49, 50^ and also on water productivity^51, 52^, however these studies are at relatively coarser spatial scale and to our understanding none of these studies considered impact of projected climate change on WFs. Being staple food over entire subcontinent, cereal crops have major role in food security and are the backbone of the agricultural economy of India. These crops are cultivated over 100 million ha area with a total annual production of about 235.2 million tonnes^53^. Assessment of WFs of cereal crops under current and future climates has large implications on the water resources of the country. This study was planned with objectives of assessing the blue and green WF of major cereals crops in India using high resolution soil and climatic data, and to assess climate change impacts on these WFs. We considered WF assessment for paddy, wheat, maize, sorghum and pearl millet which accounts more than 98.0% of total cereals production in India.

## Scope of the study

**Figure 1:**
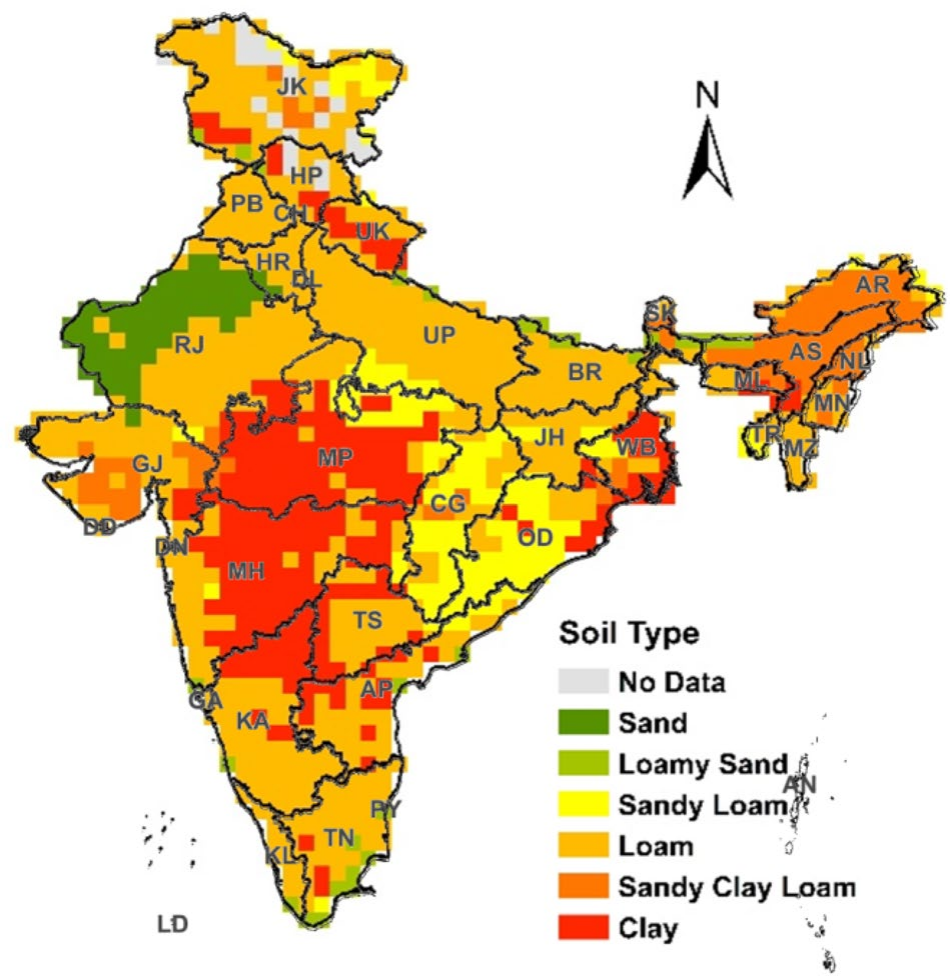
States and union territories (UT) of India with grid level soil type map derived from Harmonised World Soil Dataset (HWSD) (Spatial resolution of 0.5° latitude × 0.5° longitude equivalent to approximately 55 × 55 km grid size). The map was generated using Harmonized World Soil Database v 1.2 (http://www.fao.org) in ArcGIS 10.8.1 (https://www.arcgis.com/index.html) (State/UT codes and names: *AN* Andaman and Nicobar, *AP* Andhra Pradesh, *AR* Arunachal Pradesh, *AS* Assam, *BR* Bihar, *CH* Chandigarh, *CG* Chhattisgarh, *DN* Dadra and Nagar Haveli, *DD* Daman and Diu, *DL* Delhi, *GA* Goa, *GJ* Gujarat, *HR* Haryana, *HP* Himachal Pradesh, *JK* Jammu and Kashmir, *JH* Jharkhand, *KA* Karnataka, *KL* Kerala, *LD* Lakshadweep, *MP* Madhya Pradesh, *MH* Maharashtra, *MN* Manipur, *ML* Meghalaya, *MZ* Mizoram, *NL* Nagaland, *OD* Orissa, *PY* Puducherry, *PB* Punjab, *RJ* Rajasthan, *SK* Sikkim, *TN* Tamil Nadu, *TS* Telangana, *TR* Tripura, *UP* Uttar Pradesh, *UK* Uttarakhand, *WB* West Bengal).

This study evaluates the WFs of major cereal crops namely, paddy, wheat, maize, sorghum and pearl millet crops cultivated in India using a spatially explicit approach. These five cereal crops share 98.0% of the total cereal production in India^54^. The water use and WFs of the selected cereal crops were estimated following the methodology and terminology as described in the WF Assessment Manual^19^. The first step in the calculation of the WF of a crop production is the determination of the crop evapotranspiration which can originate either from effective rainfall (green water use) and/or irrigation (blue water use). Green water use in agriculture is the volume of evaporated rainwater by the crops while blue water use refers to evaporated irrigation water for the duration of crop growing season^18^. To account for wide spatial variation in the crop evapotranspiration, this study estimates green and blue water use of selected cereals at a spatial resolution of 0.5° latitude × 0.5° longitude. The blue and green water use of the crops was translated into the WF of crop production of the state using district level average crop yields. Figure 1 shows the 0.5° soil datasets^55^ used in this study overlaid by administrative boundaries for states/union territories in India. We also estimated the impacts of climatic change on the blue and green components of WFs of cereal crops.

## Results

### Spatial variation of WFs in future climate

**Figure 2:**
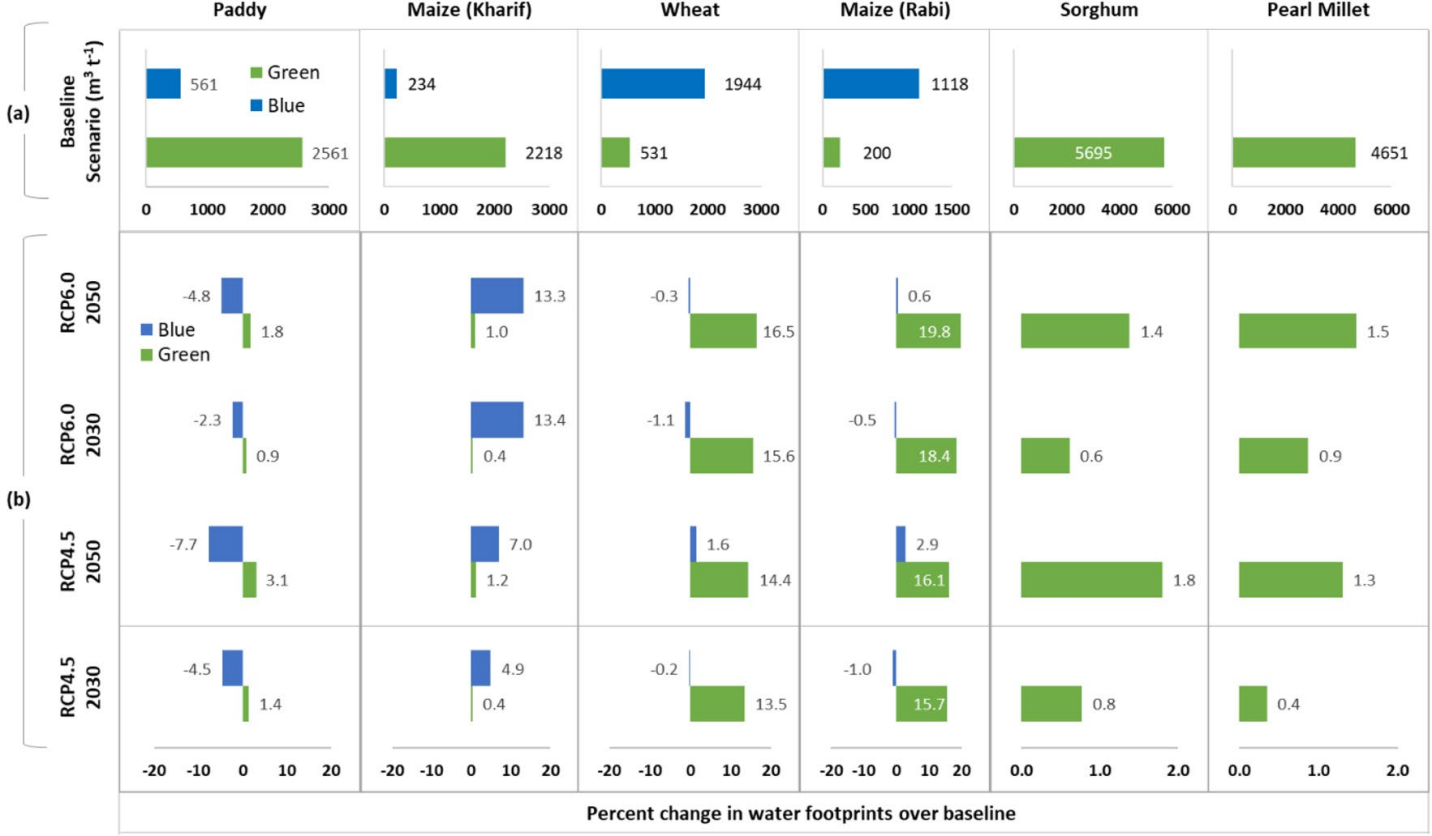
(**a**) Average blue and green WF (m^3^/t) under baseline scenario and (**b**) percent change in average blue and green WF of cereal crops under two projected climate change scenarios (RCP4.5 and RCP6.0) for the two time periods (2030s, 2050s).

The blue (or green) WF of crop production obtained by dividing the blue (or green) water use (m^3^/ha) of the cereal crops with respective crop yields (t/ha) is presented in Fig. 2a and the percent change of WFs under two projected climate change scenarios (RCP4.5 and RCP6.0) for the two time periods (2030s, 2050s) is presented in Fig. 2b. Spatial variation in the total, blue and green water use of cereal crops are presented as Fig. S1 and the temporal trends in ETc of paddy and wheat as representative kharif and rabi season crops is presented in Fig. S2 in Supplementary Material. In case of reference scenario (1989–2018), the average total WF of wheat and paddy was 2475 and 3122 m^3^ ton^−1^, respectively while the blue WF of rabi crops (wheat and rabi maize) was 2–3 time higher than that of paddy. Share of blue water in total WF of paddy, wheat, maize (kharif/rainy season) and maize (rabi/winter) were 18.0, 78.6, 95.0 and 84.8%, respectively. Higher proportion of green water (82.0–100.0%) in case of kharif/rainy season crops highlights their heavy dependence on monsoon rainfall to meet their crop water requirements. Blue WFs of paddy is projected to decrease under both the RCPs during 2030s as well as during 2050s, with a maximum decrease of 7.7% under RCP4.5 during 2050s. However, the green WFs of paddy are likely to increase (0.9 to 3.1%) under both the RCPs and future period of 2030s and 2050s. Increase in green WFs are projected to increase for all crops in both the RCPs and future period of 2030s and 2050s. The blue WFs of wheat are projected to decrease marginally with exception of RCP4.5 in 2050s. For kharif maize the blue WFs are projected increase. For rabi maize blue WFs are projected to increase marginally in 2050s and decrease marginally in 2030s (Fig. 2b).

**Figure 3:**
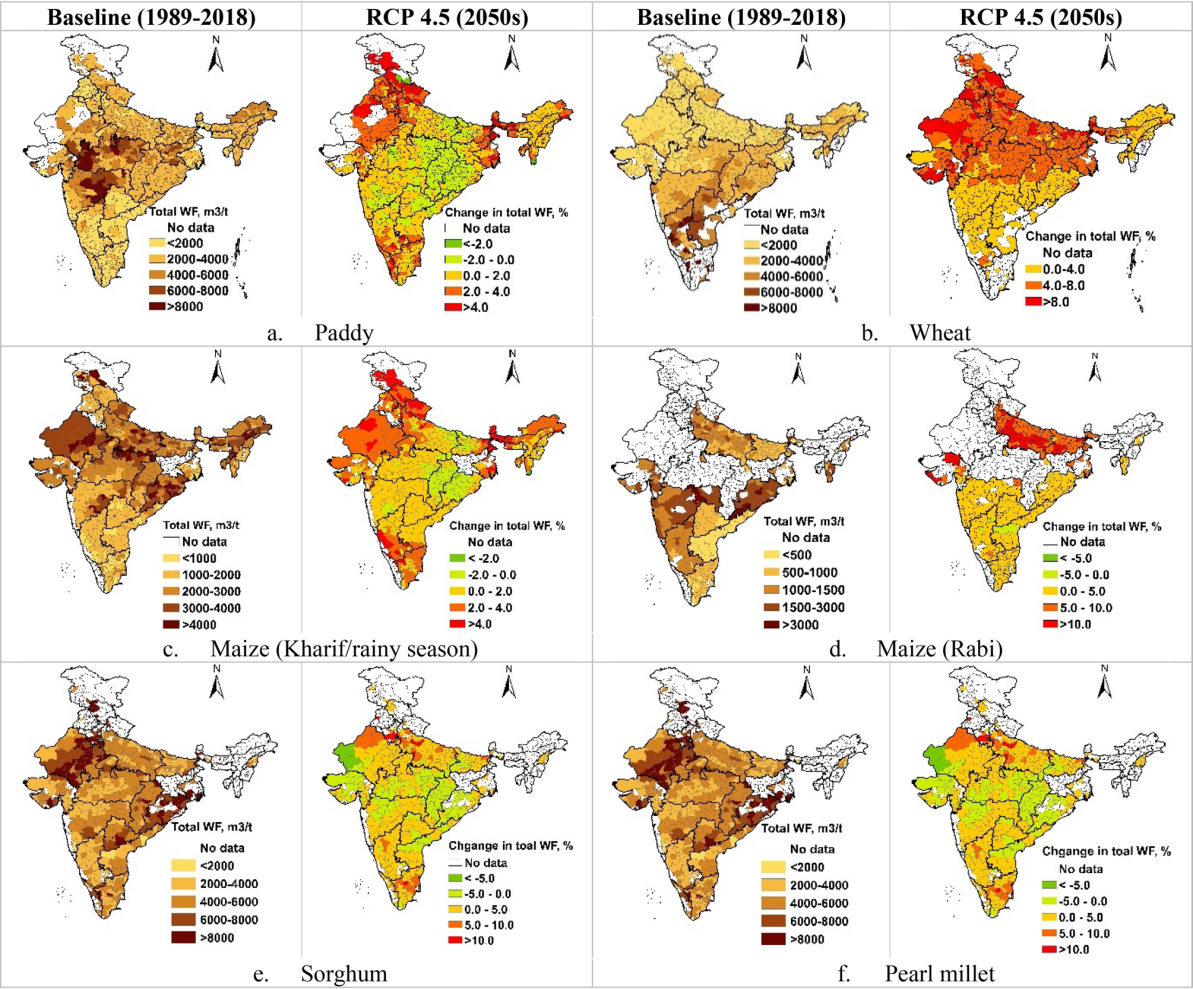
(**a–f**) Spatial variation in the total WF (m^3^/t) of crop production under baseline scenario (1989–2018) and the changes in total WF under RCP4.5 during 2050s. These maps were generated using ArcGIS 10.8.1 (https://www.arcgis.com/index.html).

As maximum change in total WF of cereal production was predicted for RCP4.5 during 2050s, we presented the spatial variation maps for total, blue and green WFs (m^3^/t) for this scenario only (Fig. 3). Previous research has also shown spatial variation of WFs of rice production under different RCPs in Nanliujiang Catchment of China^44^. Spatial variations in total and blue WFs under RCP4.5 2030, RCP6.0 2030 and RCP6.0 2050 is presented in Supplementary Material (Figs. S3–S8).

There is considerable variation in the total WFs over Indian subcontinent which is linked to spatial variation in soil and climatic condition, and crop yield differences in different sub-regions of the country. The zone of higher paddy WF (4000–8000 m^3^/t) lies in the central India covering the states of Maharashtra, Madhya Pradesh and eastern Gujrat (Fig. 3a). Total WF of kharif/rainy season maize was projected to decrease more than 4.0% in North-West India (Fig. 3c). The rabi/winter crops, however, showed higher total WF in the peninsular India. The total WF of wheat and Maize was in the ranges of 4000–8000 m^3^/t and 1500–3000 m^3^/t, respectively (Fig. 3b,d). Under RCP4.5 during 2050s, the total WF of wheat is projected to increase over the entire wheat growing region of the country with an increase of 4.0–8.0% in the northern part and 0.0–4.0% in the southern part of the country (Fig. 3b). The changes in total WF of the rainfed crops of sorghum and pearl millet under different RCPs and future time periods are marginal and would vary in the range of − 5.0 to + 5.0% over most parts of the country (Fig. 3e,f).

**Figure 4:**
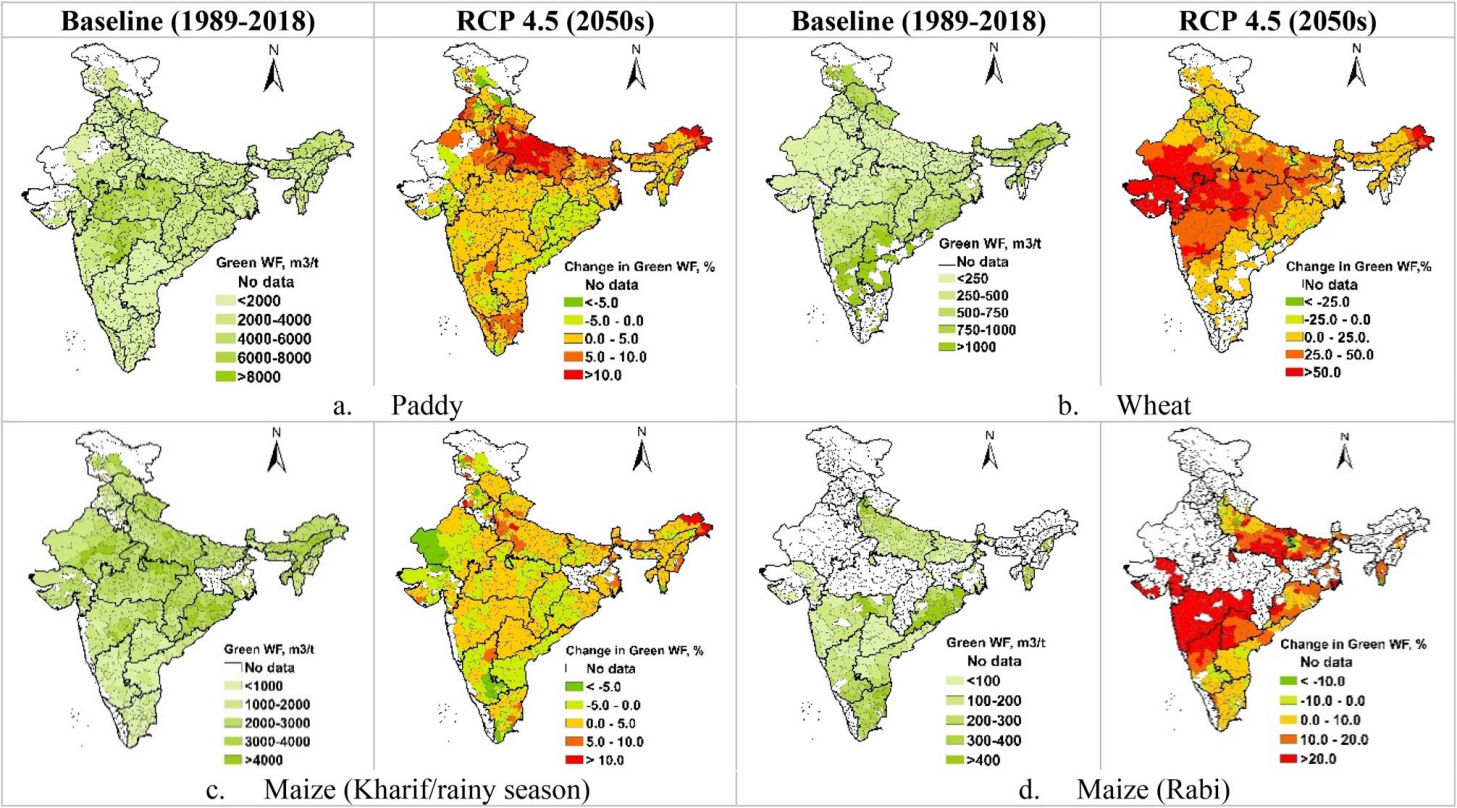
(**a–d**) Spatial variation in the green WF (m^3^/t) of crop production under baseline scenario (1989–2018) and respective percentage variations under RCP4.5 2050s. These maps were generated using ArcGIS 10.8.1 (https://www.arcgis.com/index.html).

The green WF of cereal crops is projected to increase during 2050s under RCP 4.5 (Fig. 4). The green WF of paddy is projected to increase by about 5.0% to > 10.0% in the Indo-Gangetic plains of India covering the states of Uttar Pradesh, Bihar, Punjab and Haryana, while the increase in the central region would be in the range of 0.0–5.0% (Fig. 4a). Compared to paddy, wheat and kharif season maize have lower green WFs. Projections for 2050s showed that the green WF of wheat and rabi maize would increase over larger part of the country (Fig. 4b,d). In central regions and in the western part of the country, the increase in the green WF would be to the tune of 25.0% to > 50.0% and 10.0–20.0% in case of wheat and rabi/winter maize, respectively. Kharif season maize showed 0.0–5.0% decrease in green WFs in Chhattisgarh, Odisha, Andhra Pradesh, Karnataka and parts of Gujrat and Rajasthan. The regions with increase in green WF of kharif maize were similar to that of paddy, but the increase in green WF of kharif maize is comparatively smaller (0.0–5.0%) (Fig. 4c). The green WF of rabi maize is predicted to increase by 10–20% over the major maize growing states of Maharashtra, Karnataka, Telangana, and Uttar Pradesh, however along the eastern coastal areas the increase would be in the range of 0–10% (Fig. 4d).

**Figure 5:**
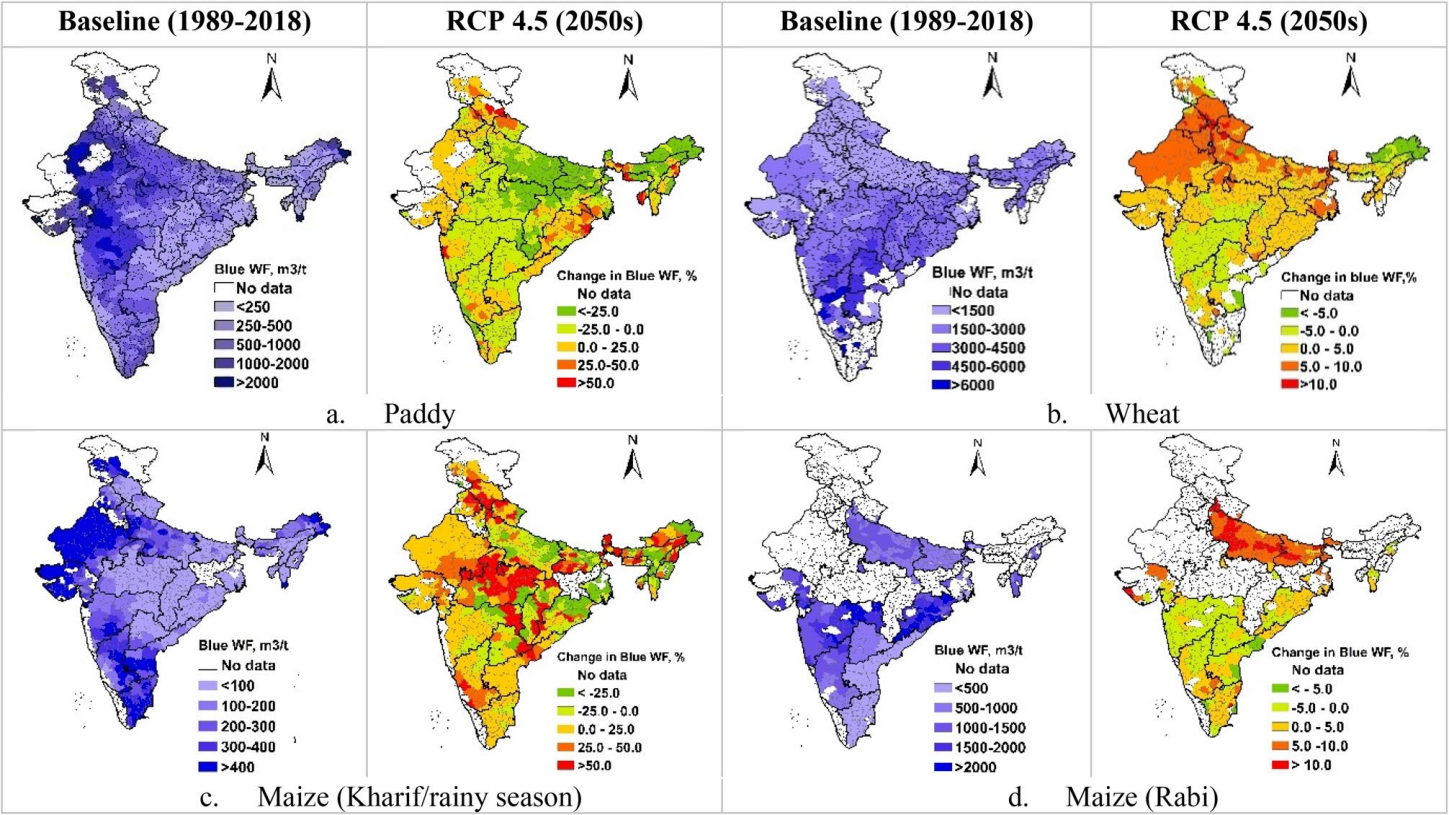
(**a–d**) Spatial variation in the blue WF (m^3^/t) of crop production under baseline scenario (1989–2018) and respective percentage variations under RCP4.5 2050s. These maps were generated using ArcGIS 10.8.1 (https://www.arcgis.com/index.html).

The regions with higher blue WF (500–2000 m^3^/t) are located in the north-western part of the country. With increase in rainfall and increased availability of green water for paddy during kharif/rainy season, there is a substantial decrease (0.0–50.0%) in blue WF in large number of paddy growing districts (Fig. 5a). Central and south-central regions of India have comparatively higher blue WFs (3000–6000 m^3^/t) of paddy mainly due to dryer winter seasons, and it is projected to increase by 5.0–10.0% over northern half of the country (Fig. 5b). Since the main source of water for rabi/winter maize is from irrigation, the blue WFs of rabi/winter maize was comparatively higher than that of kharif/rainy season maize (Fig. 5c,d). During 2050s, blue WFs of Kharif/rainy season maize is projected to increase by 25.0–50.0% or even > 50.0% over many kharif/rainy season maize growing districts of India. Blue WF of rabi/winter maize was higher (1500–2000 m^3^/t) in the eastern Maharashtra and Odisha while in the major maize growing states (Uttar Pradesh and Bihar) the blue WF was in the range of 500–1500 m^3^/t. Increase in blue WFs is projected in the range of 5.0–10.0% in Uttar Pradesh and Bihar, and in the range of 0.0–5.0% over rest part of the country (Fig. 5d). The districts located in the northern parts of the country usually have lower blue WFs of rabi/winter crops due to occurrence of winter rains and lower temperatures during crop growth period, leading to reduced irrigation water use.

### WF of cereal production under climate change scenarios

**Figure 6:**
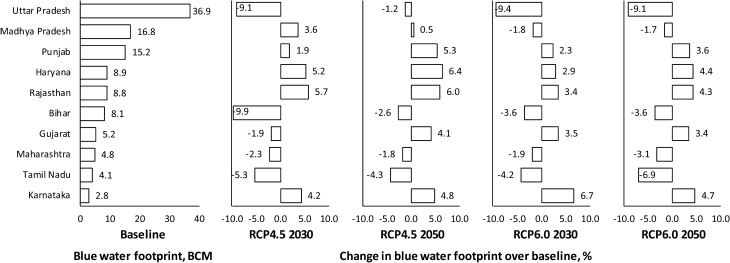
Blue WF of ten major states under baseline scenario and its variation under future climate change scenarios.

The blue WF is important in the context of management of water resources. Analysis of the state wise variation in the blue WF of cereal production in ten major Indian states for reference scenario (1989–2018) revealed that five states, namely, Uttar Pradesh, Madhya Pradesh, Rajasthan, Punjab and Maharashtra accounts for about 51.3% of the total WF of cereal production in India (Fig. 6). Similarly, five states viz. Uttar Pradesh, Madhya Pradesh, Punjab, Haryana and Rajasthan share 70.7% of blue WF of cereal production in India. The blue WF of cereal production in Uttar Pradesh, Bihar and Tamil Nadu would decrease by 9.1, 9.9 and 5.3%, respectively under RCP4.5 during 2030s, while the increase in blue WFs of Karnataka, Madhya Pradesh and north western states (Punjab, Haryana, Rajasthan) would be in the range of 1.9–5.7%. Decrease in blue WF of Uttar Pradesh is encouraging as it would relieve the pressure on surface and ground water resources, however at national scale, it is counter balanced with the increased blue WFs in other major cereal growing states (Fig. 6). State-wise and crop-wise blue and green WFs of crop production are provided in Supplementary Table S4.

As shown in Table 1, the largest share of the total WF corresponded to the green WF, accounting for 65.6% of the total WF. Total WF of cereal production under RCP4.5 during 2050s would be 365.8 BCM, about 2.6% increase over the baseline scenario. Due to increase in precipitation, the green WF showed an increase of 2.5% and 3.3% under RCP4.5 during 2030s and 2050s, respectively; and 1.1% and 1.8% under RCP6.0 during 2030s and 2050s, respectively. Our analyses showed the share of green WF in total WF would increase only marginally (0.5–1.1%) in future scenarios over the baseline scenario. Compared to the baseline, the blue WF would increase by 1.1% under RCP4.5 during 2050s, however, under RCP 6.0, it is projected to decrease by 2.2 and 2.1% during 2030s and 2050s, respectively.

**Table 1:** WF of selected cereal crops under different climate change scenarios.

Scenarios	Baseline			RCP4.5 (2030)			RCP 4.5 (2050)			RCP 6.0 (2030)			RCP 6.0 (2050)		
Crop	Total	Blue	Green	Total	Blue	Green	Total	Blue	Green	Total	Blue	Green	Total	Blue	Green
Paddy	181.2	30.1	151.1	181.6	26.1	155.5	183.2	27.3	155.9	180.4	28.0	152.4	180.9	27.4	153.5
Wheat	98.0	87.3	10.7	101.6	88.8	12.8	104.1	91.1	13.0	99.3	86.1	13.2	100.3	87.0	13.3
Maize (Kharif/rainy season)	25.0	2.9	22.0	25.0	3.1	21.9	25.5	3.3	22.2	25.2	3.5	21.6	25.3	3.5	21.8
Maize (Rabi)	2.7	2.2	0.5	2.8	2.2	0.6	2.9	2.3	0.6	2.8	2.2	0.6	2.8	2.2	0.6
Sorghum	17.3	0.0	17.3	17.3	0.0	17.3	17.5	0.0	17.5	17.3	0.0	17.3	17.4	0.0	17.4
Pearl millet	32.4	0.0	32.4	31.8	0.0	31.8	32.6	0.0	32.6	31.4	0.0	31.4	31.7	0.0	31.7
Total	356.6	122.6	234.0	360.0	120.1	239.9	365.8	124.0	241.8	356.4	119.9	236.5	358.4	120.0	238.3
% Share		34.4	65.6		33.4	66.6		33.9	66.1		33.6	66.4		33.5	66.5
% Change over baseline				0.95	− 2.04	2.52	2.58	1.14	3.33	− 0.06	− 2.20	1.07	0.50	− 2.12	1.84

Changes in total WFs of the selected cereal crops ranged from − 3.2 to 6.3% across the two RCPs and two future periods. Although, the changes in total WF were not substantial, under RCP 4.5 the blue WF of paddy would decrease from 30.1 BCM to 26.1 BCM (− 13.6%) and 27.3 BCM (− 9.6%) during 2030s and 2050s, respectively. With the increase in winter precipitation under the future climate scenarios, the green WFs of rabi/winter crops (wheat and maize) is projected to increase. Maximum increase in green WFs for wheat is projected as 24.1% under RCP6.0 during 2050s while in case of rabi/winter maize it would increase by 16.2% under RCP6.0 during 2050s. Increase in green WF of rabi/winter crops would reduce the pressure on freshwater resources. As compared to the baseline, the change in green WFs of kharif/rainy season crops viz. paddy, kharif/rainy season maize, sorghum and pearl millets would vary in the range of − 3.2 to 3.2% across all the scenarios.

## Discussion

Agriculture, owing to its heavy dependence on climatic factors, has been identified as the climate sensitive enterprise as climate change affects food production directly through changes in agro-ecological conditions^56^. The WF of crop production, the most widely used indicator of water use, is likely to be affected by the changes in climate^41, 57, 58^. The concept of WF is being used as a measure of water consumption and to identify opportunities for risk mitigation strategies that promote sustainable water use^25, 29^. In the context of wide variations in WFs of cereal crops within the Indian states, it is important to suggest crop planning such that total blue WFs can be reduced. This approach of WF based changes in cropping pattern or a drastic reduction in the irrigated areas has been demonstrated earlier^24, 25, 49, 59^. This study illustrates a comprehensive methodological framework to assess the blue and green WFs of cereal production in India considering high resolution (0.5° latitude × 0.5° longitude) assessments of crop water requirements. The baseline average blue and green WFs from this study are comparable to those found by other researchers^32, 60^.

The WF assessment methodology applied in this study is similar to earlier world-wide studies on water footprint assessment^61, 62^. However, one of the important innovations in this study is the development of integrated crop evapotranspiration (PMET) and root zone water balance (RZWB) model for the assessment of blue and green crop water footprints. The model provides a robust framework for WF assessment of agricultural crops at desired spatial resolution and can be run in the MS Excel environment using large spatial datasets pertaining to soil types, climate and crop data. On the account of absence of suitable model, earlier studies have assessed the WFs of crops at coarser resolutions. Some studies considered administrative boundaries, such as state^32, 63^ and country^60, 64^ as homogeneous unit in the assessment of water footprints. Researchers evaluated water footprints of specific crops considering grid based spatial resolutions of 30 × 30 arc minutes^65^, 5 × 5 arc minutes^66^. One limiting factor of these studies is the use of global database inputs of climate factors for ET calculations with coarser resolution, thereby limiting the accuracy of the results^67^. The modular and spatially explicit methodology developed in this study has the potential to be replicated for other agro climatic regions at any desired spatial resolution, provided the soil, climate and crop related datasets are available at the selected resolution.

Uncertainties in climate change projections remain particularly high, and combined with economic and political drivers of change, they make local level effects difficult to predict^68^. Consideration of multiple climate projections allows for consideration of various parametric, structural, and forcing uncertainties^69^. To address the uncertainties associated with GCMs and emission scenarios in climate change impact assessment, we considered multi-model ensemble of GCMs. Climate change projections of two RCPs representing intermediate stabilization pathways and two future time periods, covering wide spectrum of possible scenarios in WF assessment were used. Keeping in view the uncertainty associated with the future crop water requirements under various climate change scenarios^70^, the results obtained in the study should be interpreted considering the consequential inherent uncertainties.

In general, it is difficult to attribute differences in WF estimates across studies since they depend on a large set of assumptions on datasets, modelling structure and parameters^71^. The total WFs of wheat, maize, sorghum and pearl millet obtained in this study were slightly higher while the total WF of paddy was slightly lower than those reported by Kampman^32^. These variations can be accorded to the differences methodology and data used, and the spatial resolution of assessments. Kampman^32^ used state level average ET_c_ values of crops to compute the WF of crop production in India. The present study uses high resolution climatic and soil data to account for regional variations in soil and climate. The average WF of paddy (3122 m^3^/t) and wheat (2475 m^3^/t) worked out in this study were higher than the respective global averages of 1450 and 1830 m^3^/t^71^. Worldwide, the WF of wheat varied between 566 (United Kingdom) and 3710 m^3^/t (Morocco)^71^, while in case of for paddy it was in the range of 638 m^3^/t (Vietnam) to 2874 m^3^/t (Pakistan)^61^. Higher WFs of paddy and wheat in present study can be accorded to the differences in global and Indian average yields of these crops. The global average yields of paddy and wheat are 3.3 t/ha and 4.6 t/ha as against the Indian national average yields of 2.6 t/ha and 3.5 t/ha, respectively. Keeping other factors same, low crop yields lead to higher WFs of crop production. It is to be noted that green water covers about 2/3rd of total WFs and is comparable with estimated consumptive use of water in case of most of the cereal crops and soybean^72, 73^.

Climate change studies indicated that the inter-annual variability of the monsoon is expected to increase in the future due to possible climate change with increasing/decreasing trends at some locations^74, 75^. Climate linked variability in rainfall patterns has led to spatial variations in the WFs of crops over Indian subcontinent. Rainwater availability is seen to decrease and the temperatures increase with a delay in the sowing dates in the futures scenarios causing an increase in the crop water requirements^56^. The present study confirmed that, the future variations in rainfall and temperature patterns under future scenarios (RCP 4.6 and RCP6.0) are likely increase the crop water requirements leading to higher green and blue WFs of some regions in future climates. Reduction in blue water use over major cereal growing state (Uttar Pradesh) is predicted under all future scenarios. This is mainly on account of reducing trends observed in the annual as well as monsoon season rainfall^76^.

Climate change projections showed that under RCP4.5 during 2030s and 2050s, the blue WF of wheat and rabi/winter maize would increase by 4.4 and 13.2%, respectively. Increase in blue WFs implies increased use of surface and groundwater resources under future climates. This has direct bearing on sustainability of groundwater resources, particularly during rabi/winter season, as rabi/winter crop production is heavily dependent on regional aquifers^77^. If blue WF exceeds the availability of blue water, human water use is met by using environmental flows, leading to river and groundwater degradation^39^. This calls for optimal crop planning to minimise national blue WFs and reorganization of cropped areas in accordance with region water resource availability and crop water footprints can be a better alternative^49^. However, relocation of crop production is largely governed by social and economic factors in addition to saving in blue WFs. Predictions also showed only marginal increase (3.3 and 1.8% under RCP4.5 and RCP6.0) in the green water footprints. Increase in green WF has close links with the environmental flows in the streams and rivers. Increased green water consumption may reduce environmental flows below the threshold level required to maintain healthy aquatic ecosystems.

Ever increasing economic and population growth coupled with climate change is leading to increased water scarcity in many parts of the world. Agriculture being the most water consumptive sector and increasing international trade of agricultural commodities, the freshwater issues need to be regulated by sustainable policies. Formulation of import–export policies in India should consider the regional variations in water footprints with export restrictions on hot-spot areas facing water sustainability issues. Export of water intensive crops should be from the states where the blue WFs are lower and that the net gain from the international trade leads to positive virtual water balance.

Although, this study provides improved estimates of WF through high resolution assessments, the accuracy of assessments is largely influenced by the input data used and other assumptions. Consideration of uniform sowing dates, agronomic management practices, and static crop yield in the future in the monsoon dependent agricultural production systems can result in some deviations from actual values. On account of lack of regional values, the Kc values for a particular crop were assumed to be uniform over the Indian subcontinent as considered in previous studies^32, 38, 72^. However, Kc values differ only marginally from region to region and consideration of uniform Kc values over India, would not lead to significant variations in the WF estimates. Due to lack of accessible country wide data on agricultural pollution, we do not consider grey WFs in this study. The analysis here develops on current yield levels and we did not consider any yield changes (both positive and negative) in future climate in addition to integrated effects of increased CO_2_ on Crop Water demand. The WF estimation has been done in decoupled fashion and it has not been linked to hydrological models to understand its effect on surface water flows and change in ground water storages. Nevertheless, the impact analysis presented in this study clearly highlights the degree of expected changes in WFs under future climates.

## Conclusions

Water footprint (WF), a comprehensive indicator of water resources appropriation, is being used as a decision support tool to identify risk mitigation strategies to promote sustainable water use. This study presents a comprehensive methodological framework to assess the WFs of cereal production in India considering high resolution (0.5° latitude × 0.5° longitude) assessments of crop water requirements. The modelling framework consisted of Evapotranspiration based Irrigation Requirement (ETIR) tool, consisting of two modules, namely, PMET- for estimation of ET_0_ and RZWB- for simulating the root zone water balance. The daily crop evapotranspiration and irrigation water requirement were computed for 1204 grid points (0.5° latitude × 0.5° longitude) covering the study domain, and then aggregated to district level using weighted area proportionating approach. For assessing climate change impact on WFs, multi-model ensemble climate change scenarios were generated using the hybrid-delta ensemble method for two different RCPs (RCP4.5 and RCP6.0) and future periods of 2030s (2020–2049) and 2050s (2040–2069).

This study demonstrated that projected climate change is likely to modify the WF of cereal production in India considerably both positively (i.e., with increased green water use and reduced irrigation needs), and negatively (i.e., with reduced green water use and increased irrigation needs). Compared to the reference scenario, total WF of the selected cereal crops is projected to change in the range of − 3.2 to 6.3% across the two RCPs and two future periods. There is considerable spatial variation in the total WFs of cereal crops over the Indian subcontinent. Concentration of large proportion (51.3%) of the total WF of cereal production in five states (Uttar Pradesh, Madhya Pradesh, Rajasthan, Punjab and Maharashtra) highlights the need for devising region-specific crop planning such that total blue WFs can be reduced. Under RCP4.5 the blue as well as green WF of rabi cereals are likely to increase by 2030s and 2050s. Predictions for RCP6.0 showed that the green WF of wheat and rabi maize is projected to increase by 24.1% and 16.2% while the blue WF of wheat and rabi maize is projected to decrease (0.1–0.3%). Reduction in blue WF implies reduced pressure on blue water resources during rabi/winter season. The methodological framework presented in this paper for assessing the green and blue WFs of crops provides insights into influence of climatic factors on future trends in WF of cereal production in India. This study clearly demonstrated that water resource management strategies and policies should consider the crop-wise variations in future water use and highlighted the need for developing region specific adaptation plans considering climate change impact on WFs.

## Data and methodology

### WF estimation under current and future climate

The WF of crop production depends on the crop water consumption (including blue and green water) over the crop growing period and the crop yield^19^. Variability of climatic factors would cause the variation of crop evapotranspiration (ET_c_), irrigation water requirements (I_r_) and will exert an indirect impact on the WF of cereal crops. In this study, the reference evapotranspiration (ET_0_), crop evapotranspiration (ET_c_), effective rainfall (P_eff_) (green water use) and irrigation water requirement (blue water use), under different climatic scenarios, were determined using Evapotranspiration based Irrigation Requirement (ETIR) tool. The ETIR was developed in MS Excel using Visual Basic Applications (VBA) of Excel.

The ETIR has two modules, PMET for estimation of ET_0_ and RZWB for simulating the root zone water balance. For each of the 1204 grid points within the modelling domain, the PMET module estimated the reference evapotranspiration using the FAO-56 Penman–Monteith (PM) method^78^ and the RZWB module simulated the root zone water balance to work out the blue and green water use of selected crops at daily time step for the length of period specified by the user (30 year in this study). Daily maximum and minimum temperature, latitude and altitude of the grid point (or location) are inputs to the PMET module. Other input parameters to PM method viz. humidity, radiation, wind speed were estimated using the inbuilt functions as specified in FAO-56^78^, Najmaddin et al.^79^ and Zotarelli et al.^80^. The daily values of parameters like, soil heat flux density (G), saturation vapour pressure (es), actual vapour pressure (ea), slope of the vapour pressure versus temperature curve (Δ), solar radiation flux density at the surface (Rs), net shortwave radiation flux density (Rns), extraterrestrial radiation (Ra), inverse of the relative distance between the Earth and the Sun (dr), net longwave radiation flux density (Rnl), clear-sky solar radiation flux density (Rso) and psychrometric constant (γ) were estimated using the set of empirical equation coded in MS Excel. In PMET tool uses these set of equations for estimating all these parameters at daily time step. The details of the ETIR tool and the step-by-step modelling procedure are provided in supplementary material. During validation, the developed model showed good degree of agreement with CROPWAT estimated values of ET_c_ and P_eff_ with R^2^ values of 0.90 for ET_c_ and 0.96 for effective rainfall (P_eff_). The respective values of root mean square error were 22.8 and 27.1 mm. To get the district level averages of ET_c_ and P_eff_, the model estimated values at 0.5° latitude × 0.5° longitude were aggregated by averaging these parameters for all the pixels within the district and border pixels with > 50.0% area within the district.

**Figure 7:**
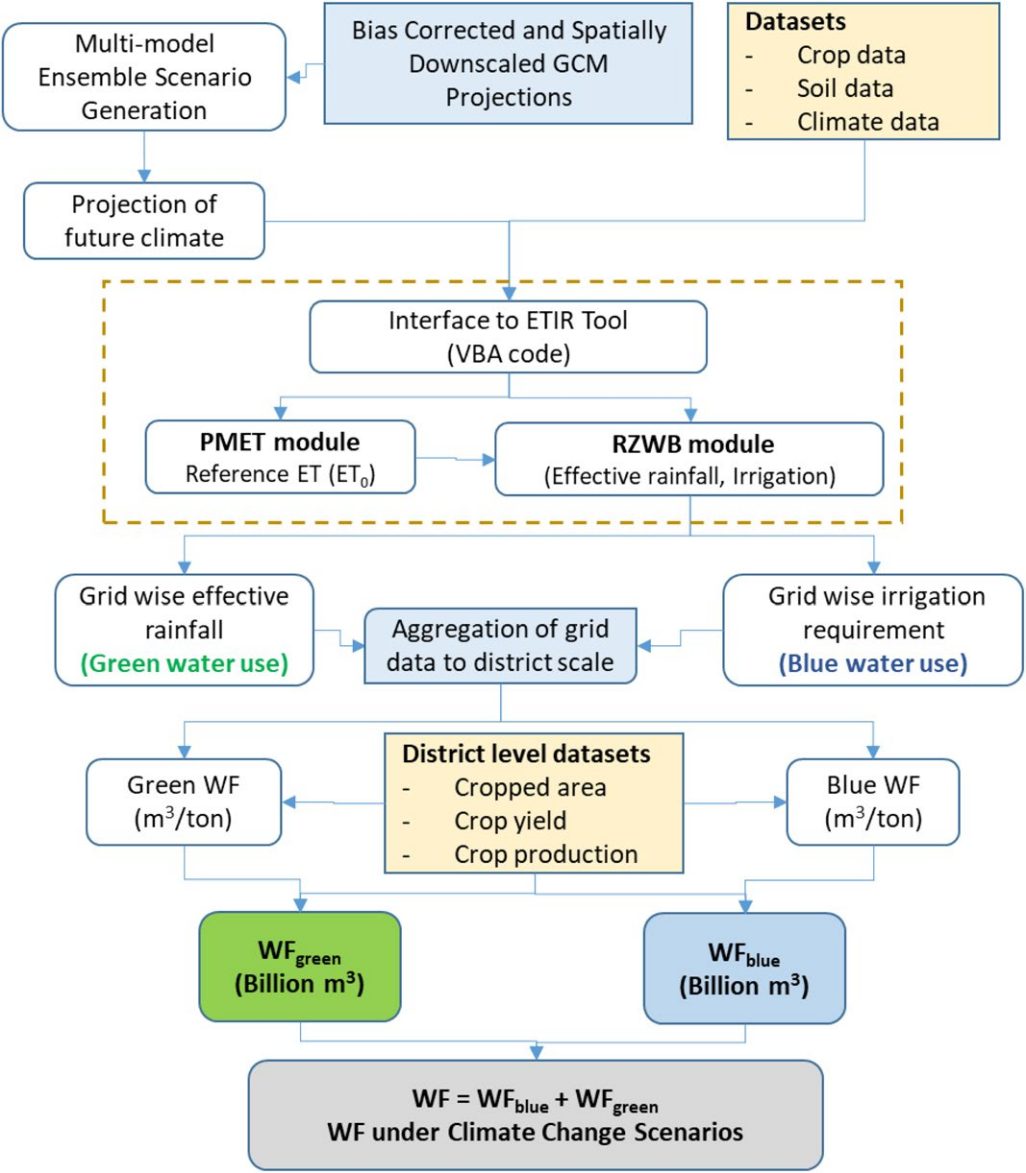
Methodological framework for the assessment of climate change impact on the WF.

The period of 1989–2018 was considered as the reference period for comparison of WFs estimated for future climate change scenarios (2030s and 2050s).We used hybrid-delta ensemble method^81–83^ for generation of climate change scenarios from multiple GCM projections for two different RCPs (RCP4.5 and RCP6.0).The detailed flowchart of WF calculation is shown in Fig. 7 and datasets used for WF assessment are provided in Table 2. Details about GCM projections used for generating climate change scenarios, crop data and soil physical properties are provided as Supplementary Material (Tables S1–S3).

**Table 2:** Datasets used in the study.

Data	Description and source
Crop area and yield	Publicly available data from the Government of India, available here (https://eands.dacnet.nic.in/)
Crop coefficients, phase duration and planting dates	Kc values for wheat, sorghum and pearl millet were adopted from^85^ while those for maize and paddy were taken from FAO-56^78^. Sowing and harvesting dates for selected cereal crops were primarily assimilated from Ministry of Agriculture^86^ and from research bulletin^87^
Soil data	Harmonized World Soil Dataset (HWSD), Version 1.2^55^ of the Food and Agriculture Organization (FAO), Rome
Soil properties: field capacity, wilting point and maximum infiltration	Estimated using pedotransfer functions for the Indian soils developed by Adhikary et al.^88^ and further validated with data presented in Raychaudhuri et al.^89^
Metrological data	High-resolution (1° latitude × 1° longitude) daily gridded temperature data^90^ and 0.25° latitude × 0.25° longitude gridded daily rainfall data^91^ for the period for the period 1989–2018 over Indian region was obtained from India Meteorological Department (IMD) Pune, India
Climate change projections	Bias corrected and spatially disaggregated (BCSD) monthly projections at 0.5° latitude × 0.5° longitude resolutions from the World Climate Research Program’s (WRCP’s) Coupled Model Inter-comparison Project phase 5 (CMIP5) multi-model dataset for the period 1950–2099^92^

The WF of cereals is the volume of water required to produce one unit of cereal grains, generally expressed in terms of cubic metres of water per ton. The total, blue and green WF of crops was obtained by dividing the district level average crop yields (Y) by the ETIR estimated district level average blue and green water use^19^.

1 $${WF}_{b/g}=\frac{{CWU}_{b/g}}{{Y}_{c}},$$

2 $${WF}_{c}={WF}_{blue,c}+{WF}_{green,c},$$

where, ${\mathrm{WF}}_{\mathrm{b}/\mathrm{g},\mathrm{c}}$ is the blue (or green) WF of the crop c (m^3^/t), ${\mathrm{CWU}}_{\mathrm{b}/\mathrm{g},\mathrm{c}}$ is the blue (or green) and green water use of crop cin district d (m^3^/ha) and ${\mathrm{Y}}_{\mathrm{c}}$ is the yield of crop c in in district d, (t/ha).

The blue and green WFs were quantified following the approach of Mekonnen et al.^71^ and Hoekstra and Mekonnen^84^. The total WF related to cereal crop production was estimated by multiplying WF of crop (unit weight basis, m^3^/t) with its total production (ton) within the district and then summing up for all districts to get the national or state level WFs.

## Supplementary Information

Supplementary Information.
