# Supplementary material for: A high-resolution assessment of climate change impact on water footprints of cereal production in India
Source: Sci Rep. 2021 Apr 22;11:8715. doi: 10.1038/s41598-021-88223-6 (PMC8062457; doi:10.1038/s41598-021-88223-6)
Supplement: Supplementary file 1 — Supplementary Information. [file 41598_2021_88223_MOESM1_ESM.docx]

**A High-Resolution Assessment of Climate Change Impact on Water Footprints of Cereal Production in India**

S. S. Mali, Paresh B Shirsath and Adlul Islam

#### Supplementary Material

#### Evapotranspiration based Irrigation Requirement Tool (ETIR Tool)

The ETIR has two modules:

1. PMET- for estimation of ET_0_

2. RZWB- for simulating the root zone water balance.

1. **PMET Module**

The PMET tool uses the well-known Penman-Monteith equation (Eq-1) to work out the reference evapotranspiration at each grid point. For each of the 1204 grid points within the modelling domain, the PMET module estimated the reference evapotranspiration using the FAO-56 Penman-Monteith (PM) method ^1^ and the RZWB module simulated the root zone water balance to work out the blue and green water use of selected crops at daily time step for the length of period specified by the user (30 year in this study). Daily maximum and minimum temperature, latitude and altitude of the grid point (or location) are inputs to the PMET module. Other input parameters to PM method *viz.* humidity, radiation, wind speed were estimated using the inbuilt functions as specified in FAO-56 ^1^ and Zotarelli et al. (2010)^2^.

$${ET}_{0}=\frac{0.408\Delta\left( R_{n}-G \right)+\gamma\frac{900}{T+273}u_{2}(e_{s}-e_{a})}{\Delta+\gamma(1.034u_{2})}$$

………………. (1)

Where, *ET_0_*= reference evapotranspiration rate (mm d^-1^), *Rn*= net radiation at the crop surface (MJ m^-2^ day^-1^), *G=* soil heat flux density (MJ m^-2^ day^-1^), *T*= mean daily air temperature at 2 m height (°C), *u_2_* wind speed at 2 m height (m s^-1^), *e_s_*= saturation vapour pressure (kPa), *e_a_*= actual vapour pressure (kPa), e_s_ - e_a_= saturation vapour pressure deficit (kPa), Δ= slope vapour pressure curve (kPa °C^-1^), *g*= psychrometric constant (kPa °C^-1^).

1. **RZWB module**

The ETIR tool imports the PMET estimated daily *ET_0_* values into the RZWB module to assess the crop water requirements and simulates available water in the crop root zone at daily time step. This one-dimensional model allows for rainfall (*P*) and reference evapotranspiration (*ET_0_*) as inputs and gives runoff (*R*), irrigation requirement (*I_r_*) and deep percolation (*DP*) as output. The available water on a day (*AW_t2_*) is estimated from the available water on the previous day (*AW_t1_*) as follows.

${AW}_{t2}={AW}_{t1}+\left( P_{t2}+{I_{r}}_{t2} \right)-\left( {{ET}_{c}}_{t1}+R_{t1}+{DP}_{t1} \right)$……….… (2)

Where $P_{t2}$and ${I_{r}}_{t2}$ are daily rainfall and irrigation (mm); ${{ET}_{c}}_{t1}$ is crop evapotranspiration (mm); $R_{t1}$ is runoff (mm) and ${DP}_{t1}$ is deep percolation (mm) beyond the root-zone. The subscripts _t1_ and _t2_ indicates the day-1 and day-2, respectively.

The model assumes that lateral inflows are balanced by outflows and there is no upward flux from a shallow water table. It is a point model, so outputs of deep drainage and runoff cannot be directly scaled up to the regional scale. Soil properties namely, saturation percentage (SP), field capacity (FC), wilting point (WP) and deep percolation rate (DP) needs to be specified for each soil type. For each year, the ETc is calculated for the cropping duration using the ET_0_ and Kc values of respective growth stage. In estimating the irrigation water requirement, the crop specific management allowed depletion (MAD) (certain percentage of (FC-WP)) needs to be specified. The ETIR tool schedules an irrigation event when the soil water deficit (FC-AW_t2_) in the root zone drops below the MAD. Assessment of irrigation water requirement can be done using either ‘refill to field capacity’ option or ‘application of specified depth’. The model estimates irrigation requirement on a day (*I_rt2_*) on the basis of soil water content of previous day (*SWC_t1_*) (eq.3).

*I_rt2_ = FC-SWC_t1_* ……... if SWC*_t1_* < WP+(100-MAD) * (FC-WP) … (3)

If on a particular day (*_t2_*), the total soil water is greater than the FC of the soil, deep percolation starts from the root zone. The amount of deep percolation (*DP_t2_*) is adjusted as per the amount of extra soil water content above the FC of soil as.

*DP_t2_ = DP_max_ … if SWC _t1_ ≥ FC+DP_max_* ...(4)

*DP_t2_ = SWC_t2_-FC ... if FC< SWC_t1_ ≤ FC+DP_max_* … (5)

Where, *DP_t2_* is the deep percolation rate on day 2, *DP_max_* is the maximum infiltration rate of the soil, *SWC_t1_* is the soil water content on previous day (day 1).

In case the AWas estimated using eq. 3 exceeds the saturation capacity (*WC_sat_*) of the soil, the amount of water exceeding the *WC_sat_* is accounted as runoff (*R*). Generally, paddy fields are bunded and allow ponding to a depth ~50 mm ^3^. Runoff from paddy fields starts after the ponding requirement is met. To account this consideration, depth of ponding (*D_pond_*) is added to the saturation capacity of the soil to account for delayed runoff generation on account of field bunds. Runoff on day 2 (*R_t2_*)is estimated as;

*R_t2_= AW_t2_ - WC_sat_+D_pond_* ...(6)

On every rainfall event the soil moisture content gets updated as per the water holding capacity (WHC) of the soil implying postponement of irrigation until SWC drop below MAD. The seasonal effective rainfall (*SP_eff_*) and seasonal irrigation water requirement (*SI_r_*) were obtained as:

*SP_eff_= SP-SR-SDP* … (7)

*SIr= SETc-SP_eff_ … (8)*

Where, *SP*, *SR*, *SDP* and *SET_c_* are the rainfall, runoff, deep percolation and crop evapotranspiration during the entire crop growing period.

The VBA code was developed to access the grid point wise data pertaining to elevation, soil type, latitude, longitude, 30-year daily data on maximum temperature (*Tmax*) and minimum temperature (*Tmin*) from different excel worksheets. This data was input to the PMET module to estimate the ET_0_ for each grid point. The estimated ET_0_ was imported into the RZWB model to estimate the *ET_c_*, *I_r_* and *P_eff_* for selected five cereal crops. In case of rainfed crops (sorghum and pearl millet) the ET_green_was considered as equivalent to the total evapotranspiration, because the ET_blue_ is zero. To account for rainfed conditions, the irrigation option of ‘application of specified depth’ was selected and the irrigation depth was set equal to zero. The ETIR was used to assess the future CWR under two representative concentration pathway (RCP) scenarios (RCP4.5 and RCP8.5). The model was run separately for each crop and the grid-point wise output parameters were saved to separate worksheets and were used in developing spatial maps in GIS environment.

Data pertaining to *ET_c_*, *I_r_*and *P_eff_* of five cereal crops at 1204 grid points within India was aggregated to district level using weighted area proportionating approach. For a particular crop, the district level averages of *ET_c_* (*AvgET_c_*), *I_r_* (*AvgI_r_*) and *P_eff_*(*AvgP_eff_*) were derived using the values of these parameters at each grid points falling within the area and the area of respective grids within the district as;

${AvgET}_{c}=\sum_{i=1}^{n} \frac{A_{n}}{A_{d}}\times{{ET}_{c}}_{n}$ … (9)

Where, ${AvgET}_{c}$ is the average *ETc* of the district for crop under consideration, $A_{n}$ is the area of the n^th^ grid point within the district, $A_{d}$ is the total area of the district and ${{ET}_{c}}_{n}$ is the estimated *ET_c_* of selected crop at n^th^ grid point within the district. The weighted area averages of the *P_eff_* and *I_r_* were estimated for each of the 684 districts within India using same methodology (Eq. 9).

**References**

1. Allen, R. G., Pereira, L. S., Raes, D. & Smith, M. FAO Irrigation and drainage paper No. 56. *Rome Food Agric. Organ. United Nations* **56**, e156 (1998).

2. Zotarelli, L., Dukes, M. D., Romero, C. C., Migliaccio, K. W. & Morgan, K. T. Step by step calculation of the Penman-Monteith Evapotranspiration (FAO-56 Method). *Inst. Food Agric. Sci. Univ. Florida* (2010).

3. Balwinder-Singh *et al.* Taking the climate risk out of transplanted and direct seeded rice: Insights from dynamic simulation in Eastern India. *F. Crop. Res.* **239**, 92–103 (2019).

####

**Table S1.** GCM projections used for generating climate change scenarios

| Modeling Center or Group, Country | Model Name | Number of Runs^*^for RCP | |
| --- | --- | --- | --- |
|  |  | 4.5 | 6.0 |
| Commonwealth Scientific and Industrial Research Organization and Bureau of Meteorology (CSIRO-BOM), Australia | ACCESS1,0 | 1 |  |
| Beijing Climate Center, China Meteorological Administration (BCC), China | BCC-CSM1.1 | 1 | 1 |
|  | BCC-CSM1.1(M) | 1 |  |
| College of Global Change and Earth System Science, Beijing Normal University (GCESS), China | BNU-ESM | 1 |  |
| Canadian Centre for Climate Modelling and Analysis (CCCma), Canada | CANESM2 | 5 |  |
| National Center for Atmospheric Research, USA | CCSM4 | 5 | 5 |
| Community Earth System Model contributors, USA | CESM1-BGC | 1 |  |
|  | CESM1-CAM5 | 3 | 2 |
| Centro Euro-Mediterraneoper ICambiamentiClimatici (CMCC), Italy | CMCC-CM | 1 |  |
| Centre National de Recherches Météorologiques / Centre Européen de Recherche et Formation Avancée en Calcul Scientifique (CNRM-CERFACS), France | CNRM-CM5 | 1 |  |
| Commonwealth Scientific and Industrial Research Organization and Queensland Climate Change Centre of Excellence (CSIRO-QCCCE), Australia | CSIRO-MK3.6.0 | 10 | 10 |
| EC-EARTH consortium | EC-EARTH | 3 |  |
| LASG, Institute of Atmospheric Physics, Chinese Academy of Sciences and CESS, Tsinghua University (LASG-CESS), China | FGOALS-G2 | 1 |  |
| The First Institute of Oceanography, SOA, China | FIO-ESM | 3 | 3 |
| NOAA Geophysical Fluid Dynamics Laboratory (NOAA GFDL), USA | GFDL-CM3 | 1 | 1 |
|  | GFDL-ESM2G | 1 | 1 |
|  | GFDL-ESM2M | 1 | 1 |
| NASA Goddard Institute for Space Studies (NASA GISS), USA | GISS-E2-H-CC | 1 |  |
|  | GISS-E2-R | 5 | 1 |
|  | GISS-E2-R-CC | 1 |  |
| National Institute of Meteorological Research/Korea Meteorological Administration (NIMR/KMA), South Korea | HADGEM2-AO | 1 | 1 |
| Met Office Hadley Centre (additional HadGEM2-ES realizations contributed by Instituto Nacional de PesquisasEspaciais) (MOHC/ INPE), UK | HADGEM2-ES | 2 | 2 |
| Institute for Numerical Mathematics (INM), Russia | INMCM4 | 1 |  |
| Institut Pierre-Simon Laplace (IPSL), France | IPSL-CM5A-LR | 4 | 1 |
|  | IPSL-CM5A-MR | 1 | 1 |
|  | IPSL-CM5B-LR | 1 |  |
| Japan Agency for Marine-Earth Science and Technology, Atmosphere and Ocean Research Institute (The University of Tokyo), and National Institute for Environmental Studies (MIROC), Japan | MIROC-ESM | 1 | 1 |
|  | MIROC5 | 1 | 1 |
|  | MIROC-ESM-CHEM | 1 | 1 |
| Norwegian Climate Centre (NCC), Norway | NORESM1-M | 1 | 1 |
| Total models |  | 30 | 17 |
| Total runs |  | 61 | 34 |

*In CMIP5, ensemble members are identified by rNiMpL, where r, i, and pare realization initialization, and perturbation physics, respectively. Run reflects N from a given CMIP5 projections rNiMpL identifier

**Table S2** Crop data used in assessment of WFs of selected crops

| Crop | Sowing Date | | Duration of growth stage | | | | | | | | Crop Coefficients (Kc) | | | | | | | |
| --- | --- | --- | --- | --- | --- | --- | --- | --- | --- | --- | --- | --- | --- | --- | --- | --- | --- | --- |
|  |  |  | Initial | | Dev | | Mid | | Late | | Initial | | Dev | | Mid | | Late | |
| Maize (Kharif) | 15-Jun | | 20 | | 30 | | 35 | | 25 | | 0.55 | | 1.0 | | 1.23 | | 0.63 | |
| Paddy | 25-Jun | | 15 | | 30 | | 50 | | 25 | | 0.40 | | 1.05 | | 1.20 | | 0.85 | |
| Sorghum | 01-Jul | | 20 | | 30 | | 45 | | 30 | | 0.53 | | 0.82 | | 1.24 | | 0.85 | |
| Pearl Millet | 01-Jul | | 15 | | 25 | | 45 | | 25 | | 0.53 | | 0.82 | | 1.24 | | 0.85 | |
| Wheat | 15-Oct | 15 | | 25 | | 50 | | 30 | | 0.50 | | 1.36 | | 1.24 | | 0.42 | |  |
| Maize (Rabi) | 01-Nov | | 20 | | 30 | | 35 | | 25 | | 0.55 | | 1.0 | | 1.23 | | 0.63 | |

**Table S3** Soil physical properties considered in the root zone water balance model

| Texture | Field Capacity,  v/v % | Wilting point,  v/v % | Saturation percentage,  v/v % | Infiltration Rate,  cm/h | Deep Percolation rate, mm/day |
| --- | --- | --- | --- | --- | --- |
| Sand | 100 | 30 | 395 | 170.0 | 5.51 |
| Loamy Sand | 120 | 50 | 410 | 90.0 | 4.47 |
| Sandy Loam | 180 | 90 | 435 | 59.0 | 3.89 |
| Loam | 280 | 110 | 451 | 7.0 | 1.94 |
| Sandy Clay Loam | 350 | 200 | 420 | 20.0 | 2.73 |
| Clay | 410 | 250 | 482 | 7.0 | 1.94 |

|  | **Total water use** | **Blue water use** | **Green water use** |
| --- | --- | --- | --- |
| **Paddy** | **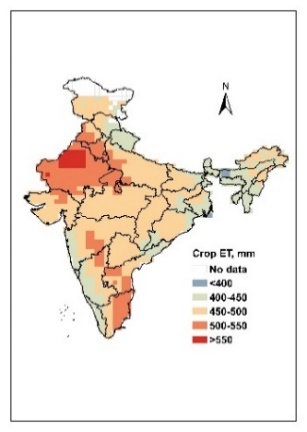** | **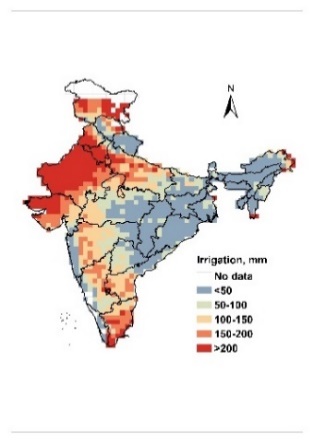** | **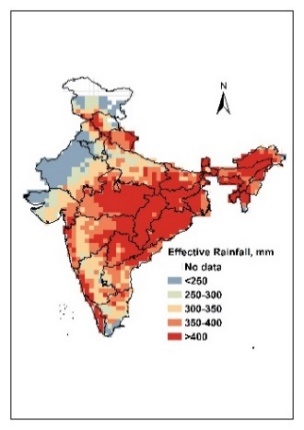** |
| **Wheat** | **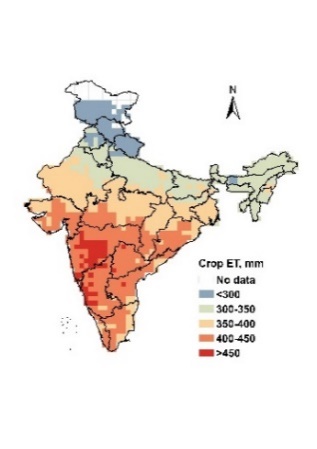** | **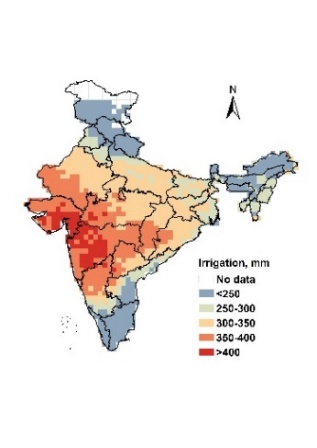** | **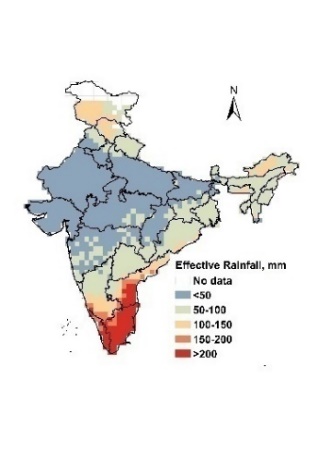** |
| **Maize (Kharif)** | **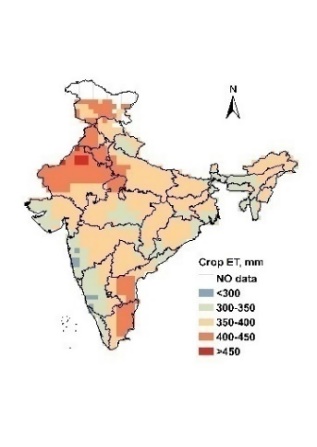** | **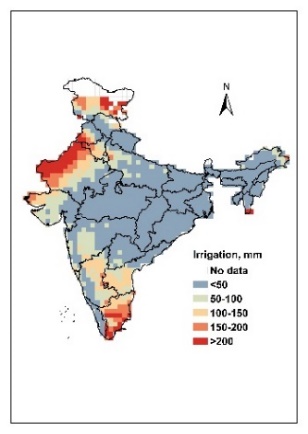** | **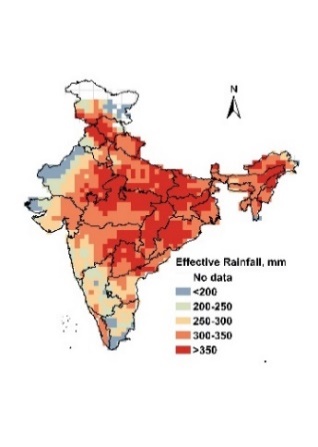** |
| **Maize (Rabi)** | **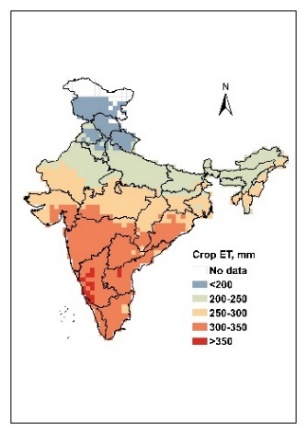** | **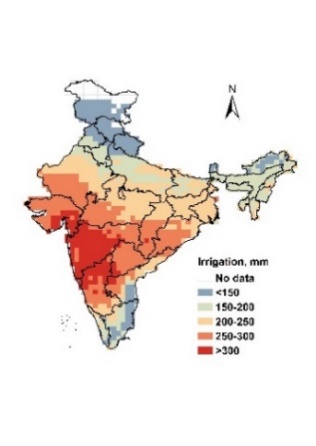** | **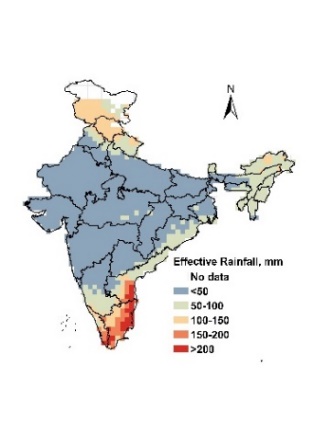** |
| **Sorghum** | **--** | **--** | **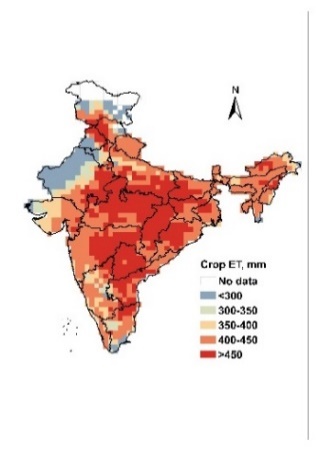** |
| **Pearl millet** | **--** | **--** | **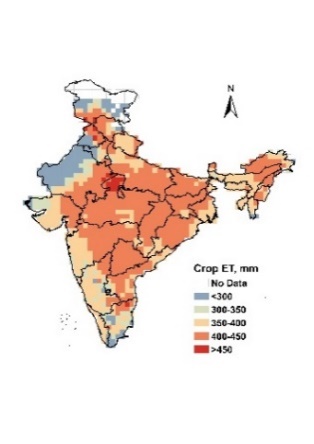** |

**Figure S1** Spatial variation in crop evapotranspiration, irrigation requirement (blue water use) and effective rainfall (green water use) of selected cereal crops under baseline scenario. These maps were generated using ArcGIS 10.8.1 (<https://www.arcgis.com/index.html>)

Table S4: State-wise and crop-wise total and blue water footprints of cereal crop production

| **State/UT** | **Paddy** | | | **Wheat** | | | **Maize (Kharif)** | | | **Maize (Rabi)** | | | **Sorghum** | **Pearl Millet** |
| --- | --- | --- | --- | --- | --- | --- | --- | --- | --- | --- | --- | --- | --- | --- |
|  | Total | Blue | Total | | Blue | Total | | Blue | Total | | Blue | Total | | Total |
| Andhra Pradesh | 7.72 | 1.04 | 0.00 | | 0.00 | 0.20 | | 0.03 | 0.37 | | 0.26 | 0.14 | | 0.22 |
| Arunachal Pradesh | 0.59 | 0.06 | 0.01 | | 0.01 | 0.17 | | 0.02 | 0.00 | | 0.00 | 0.00 | | 0.00 |
| Assam | 7.89 | 0.33 | 0.19 | | 0.14 | 0.07 | | 0.00 | 0.00 | | 0.00 | 0.00 | | 0.00 |
| Bihar | 11.00 | 1.35 | 7.43 | | 6.30 | 1.02 | | 0.02 | 0.55 | | 0.44 | 0.07 | | 0.02 |
| Chandigarh | 0.00 | 0.00 | 0.00 | | 0.00 | 0.00 | | 0.00 | 0.00 | | 0.00 | 0.00 | | 0.00 |
| Chhattisgarh | 22.20 | 1.23 | 0.54 | | 0.45 | 0.51 | | 0.00 | 0.00 | | 0.00 | 0.04 | | 0.00 |
| Dadra and Nagar Haveli | 0.06 | 0.00 | 0.00 | | 0.00 | 0.00 | | 0.00 | 0.00 | | 0.00 | 0.00 | | 0.00 |
| Goa | 0.16 | 0.01 | 0.00 | | 0.00 | 0.00 | | 0.00 | 0.00 | | 0.00 | 0.00 | | 0.00 |
| Gujarat | 3.62 | 0.90 | 4.05 | | 3.66 | 1.66 | | 0.23 | 0.44 | | 0.39 | 0.66 | | 2.81 |
| Haryana | 5.50 | 2.14 | 8.22 | | 6.74 | 0.06 | | 0.00 | 0.00 | | 0.00 | 0.48 | | 2.47 |
| Himachal Pradesh | 0.36 | 0.04 | 0.98 | | 0.61 | 1.14 | | 0.03 | 0.00 | | 0.00 | 0.00 | | 0.00 |
| Jammu and Kashmir | 1.15 | 0.43 | 0.51 | | 0.24 | 1.20 | | 0.37 | 0.00 | | 0.00 | 0.01 | | 0.07 |
| Jharkhand | 4.89 | 0.33 | 0.25 | | 0.20 | 0.00 | | 0.00 | 0.00 | | 0.00 | 0.00 | | 0.00 |
| Karnataka | 5.00 | 0.95 | 1.16 | | 0.91 | 3.03 | | 0.74 | 0.30 | | 0.23 | 1.25 | | 1.40 |
| Kerala | 0.56 | 0.04 | 0.00 | | 0.00 | 0.00 | | 0.00 | 0.00 | | 0.00 | 0.01 | | 0.00 |
| Madhya Pradesh | 8.56 | 0.82 | 18.71 | | 15.91 | 3.25 | | 0.10 | 0.00 | | 0.00 | 2.62 | | 0.84 |
| Maharashtra | 6.55 | 0.77 | 4.17 | | 3.56 | 1.58 | | 0.20 | 0.33 | | 0.27 | 6.15 | | 4.83 |
| Manipur | 0.62 | 0.08 | 0.00 | | 0.00 | 0.01 | | 0.00 | 0.00 | | 0.00 | 0.00 | | 0.00 |
| Meghalaya | 0.42 | 0.01 | 0.01 | | 0.00 | 0.06 | | 0.00 | 0.00 | | 0.00 | 0.00 | | 0.00 |
| Mizoram | 0.28 | 0.04 | 0.00 | | 0.00 | 0.04 | | 0.00 | 0.00 | | 0.00 | 0.00 | | 0.00 |
| Nagaland | 0.84 | 0.05 | 0.01 | | 0.01 | 0.22 | | 0.00 | 0.01 | | 0.01 | 0.01 | | 0.01 |
| Orissa | 15.92 | 0.91 | 0.04 | | 0.03 | 0.26 | | 0.00 | 0.05 | | 0.04 | 0.05 | | 0.02 |
| Puducherry | 0.07 | 0.03 | 0.00 | | 0.00 | 0.00 | | 0.00 | 0.00 | | 0.00 | 0.00 | | 0.00 |
| Punjab | 14.70 | 5.71 | 12.05 | | 9.44 | 0.66 | | 0.04 | 0.00 | | 0.00 | 0.00 | | 0.08 |
| Rajasthan | 0.69 | 0.27 | 9.33 | | 8.16 | 4.06 | | 0.38 | 0.00 | | 0.00 | 2.67 | | 18.54 |
| Sikkim | 0.05 | 0.00 | 0.01 | | 0.01 | 0.13 | | 0.00 | 0.00 | | 0.00 | 0.00 | | 0.00 |
| Tamil Nadu | 9.75 | 3.59 | 0.00 | | 0.00 | 0.74 | | 0.32 | 0.42 | | 0.19 | 1.40 | | 0.46 |
| Telangana | 4.34 | 0.54 | 0.04 | | 0.04 | 1.59 | | 0.10 | 0.36 | | 0.29 | 0.83 | | 0.09 |
| Tripura | 1.52 | 0.01 | 0.00 | | 0.00 | 0.01 | | 0.00 | 0.00 | | 0.00 | 0.00 | | 0.00 |
| Uttar Pradesh | 27.97 | 7.38 | 33.77 | | 29.14 | 3.21 | | 0.39 | 0.03 | | 0.03 | 1.16 | | 3.98 |
| Uttarakhand | 1.15 | 0.13 | 1.04 | | 0.78 | 0.10 | | 0.00 | 0.00 | | 0.00 | 0.00 | | 0.00 |
| West Bengal | 17.15 | 0.98 | 1.24 | | 0.97 | 0.16 | | 0.00 | 0.07 | | 0.06 | 0.01 | | 0.00 |

Figure S2. Temporal trends in the ETc of paddy and wheat in four distinct agro-ecological regions of India (baseline scenario)

**RCP4.5 2030s**

| **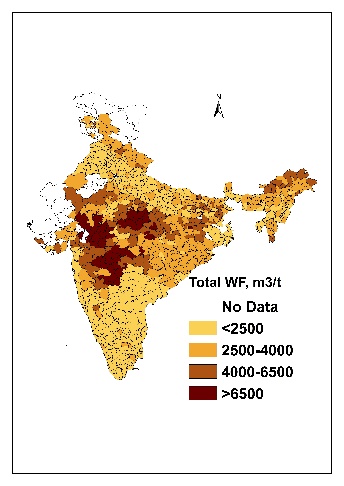** | **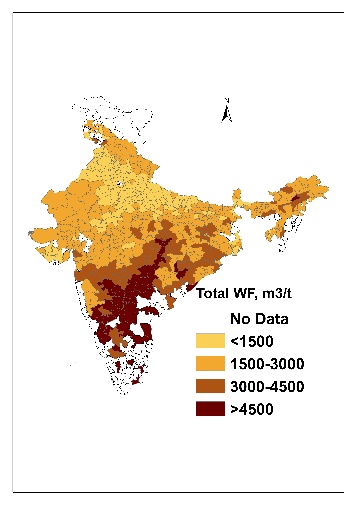** | **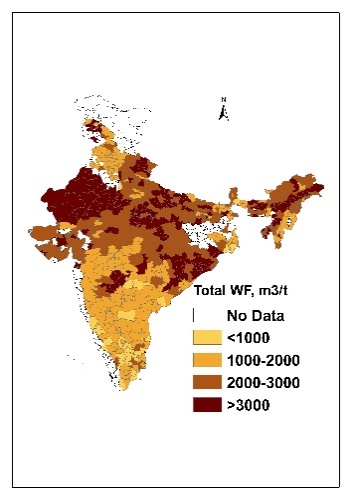** | **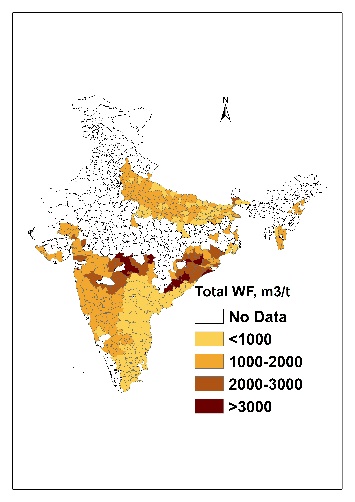** |
| --- | --- | --- | --- |
| **Paddy** | **Wheat** | **Maize (Kharif)** | **Maize (Rabi)** |
|  | **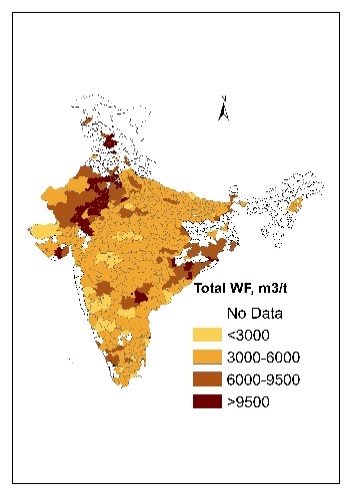** | **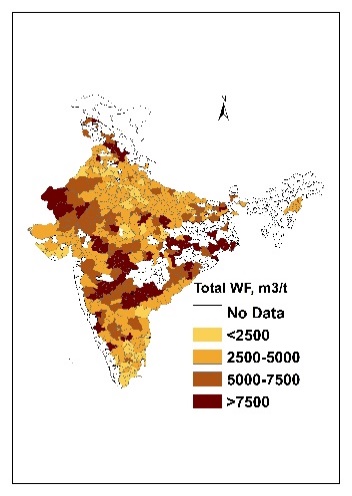** |  |
|  | **Sorghum** | **Pearl Millet** |  |

Figure S3. Spatial variation in the total WFs of crops under RCP4.5 2030. These maps were generated using ArcGIS 10.8.1 (<https://www.arcgis.com/index.html>).

| **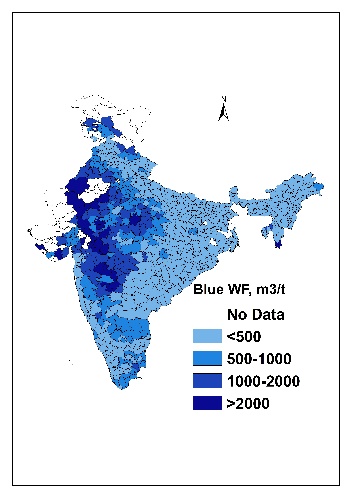** | **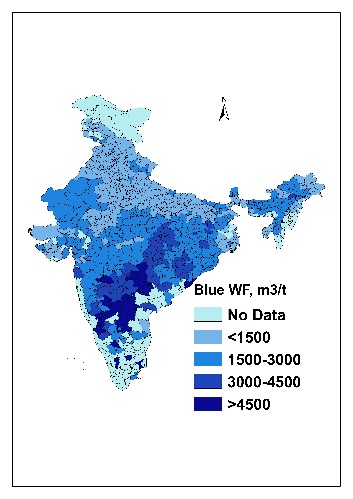** | **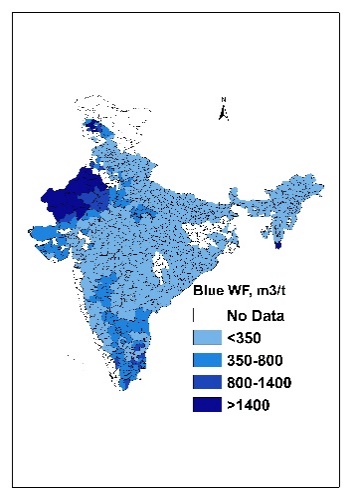** | **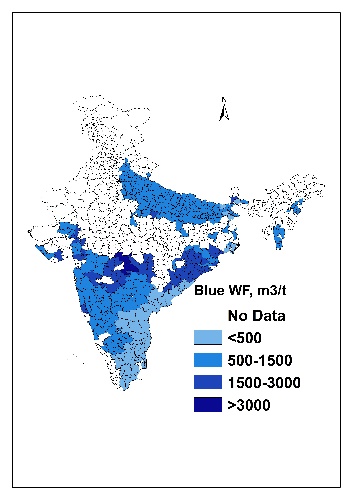** |
| --- | --- | --- | --- |
| **Paddy** | **Wheat** | **Maize (Kharif)** | **Maize (Rabi)** |

Figure S4. Spatial variation in the blue WFs of crops under RCP4.5 2030. These maps were generated using ArcGIS 10.8.1 (<https://www.arcgis.com/index.html>).

**RCP6.0 2030s**

| **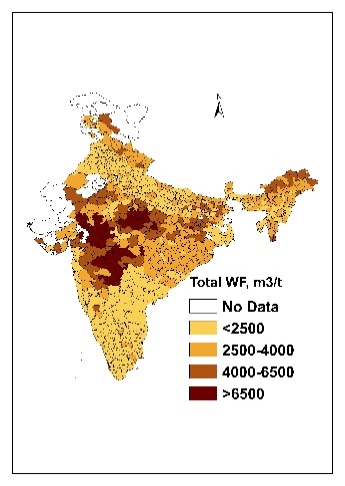** | **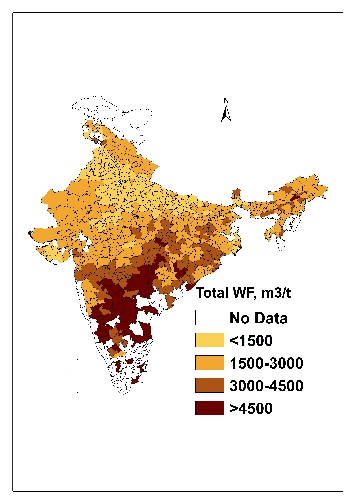** | **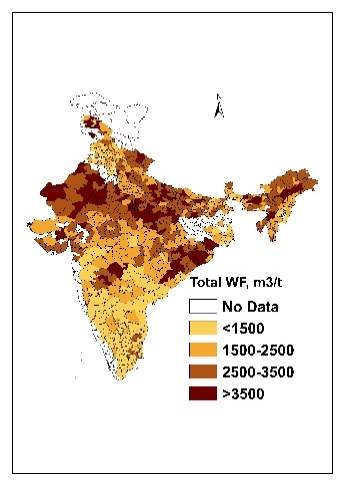** | **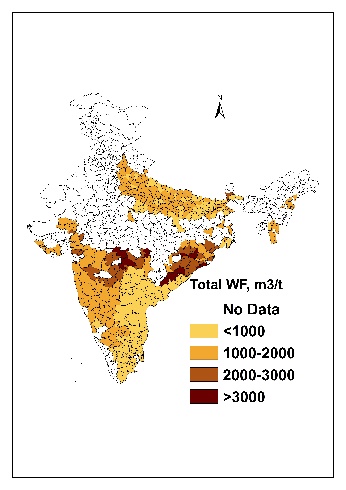** |
| --- | --- | --- | --- |
| **Paddy** | **Wheat** | **Maize (Kharif)** | **Maize (Rabi)** |
|  | **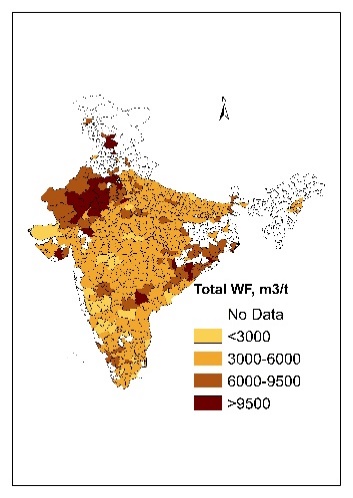** | **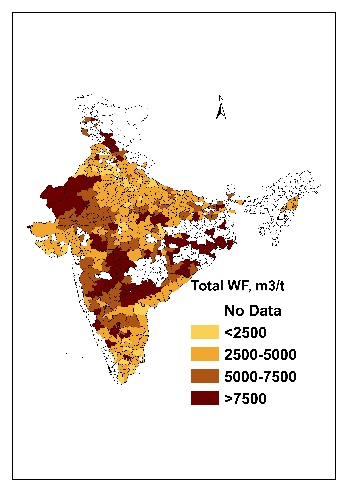** |  |
|  | **Sorghum** | **Pearl Millet** |  |

Figure S5. Spatial variation in the total WFs of crops under RCP6.0 2030. These maps were generated using ArcGIS 10.8.1 (<https://www.arcgis.com/index.html>).

| **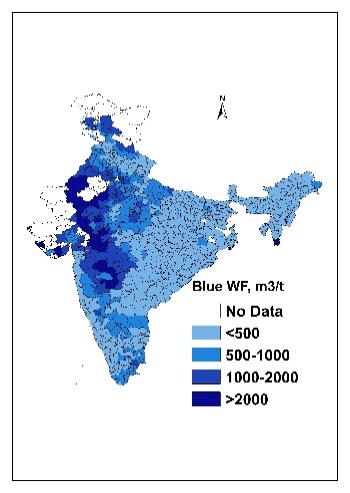** | **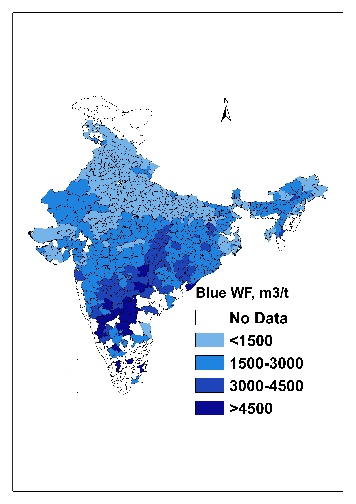** | **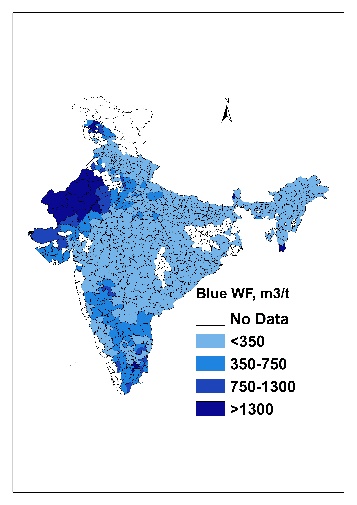** | **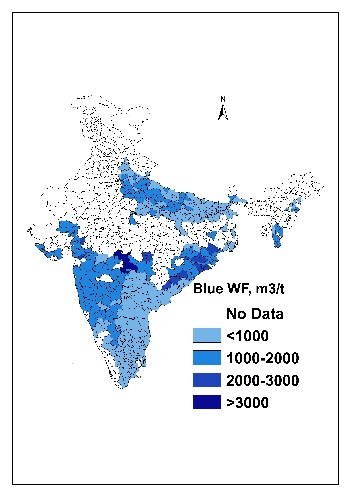** |
| --- | --- | --- | --- |
| **Paddy** | **Wheat** | **Maize (Kharif)** | **Maize (Rabi)** |

Figure S6. Spatial variation in the blue WFs of crops under RCP6.0 2030. These maps were generated using ArcGIS 10.8.1 (<https://www.arcgis.com/index.html>).

**RCP6.0 2050s**

| **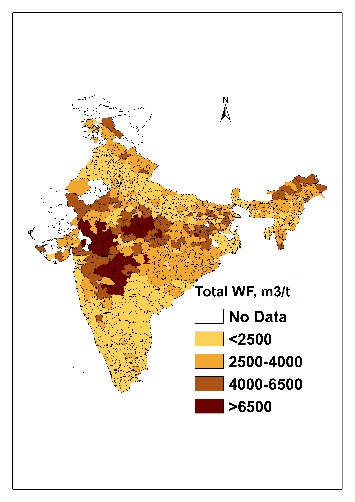** | **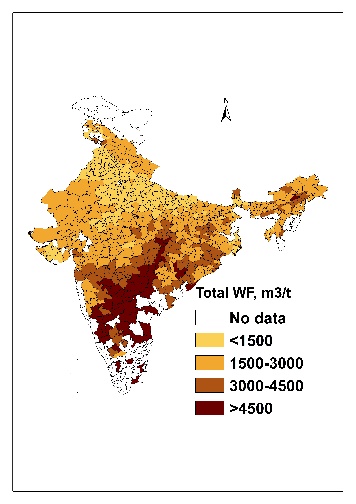** | **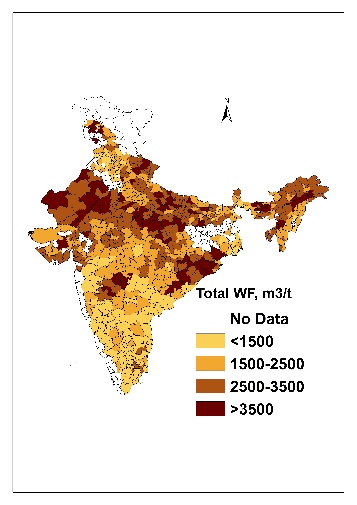** | **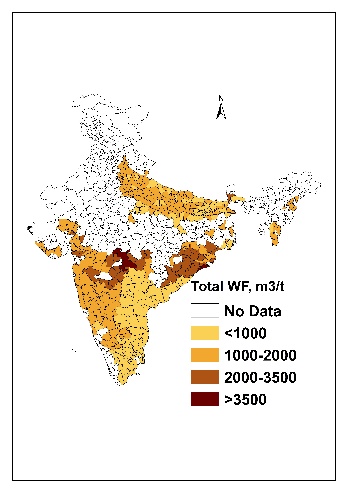** |
| --- | --- | --- | --- |
| **Paddy** | **Wheat** | **Maize (Kharif)** | **Maize (Rabi)** |
|  | **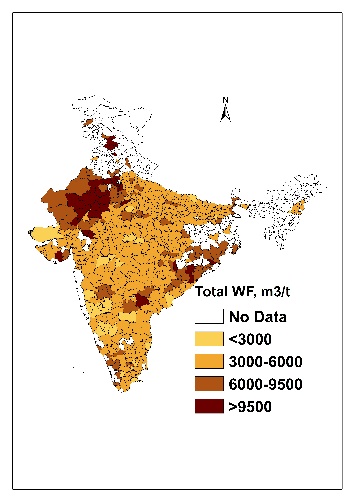** | **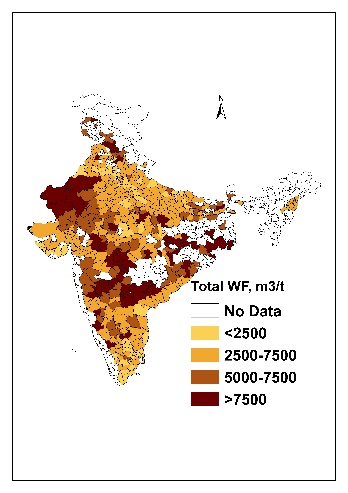** |  |
|  | **Sorghum** | **Pearl Millet** |  |

Figure S7. Spatial variation in total WFs of crops under RCP6.0 2050. These maps were generated using ArcGIS 10.8.1 (<https://www.arcgis.com/index.html>).

| **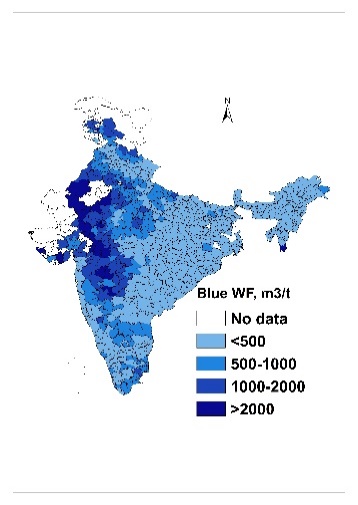** | **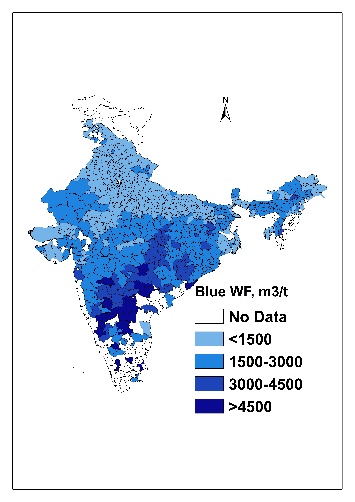** | **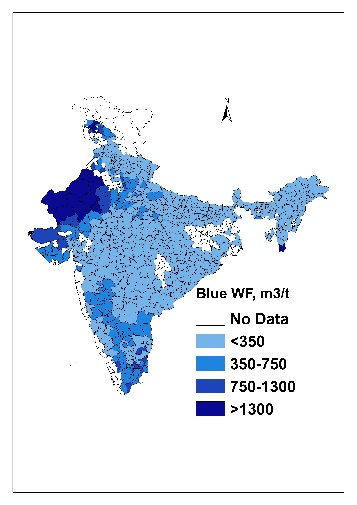** | **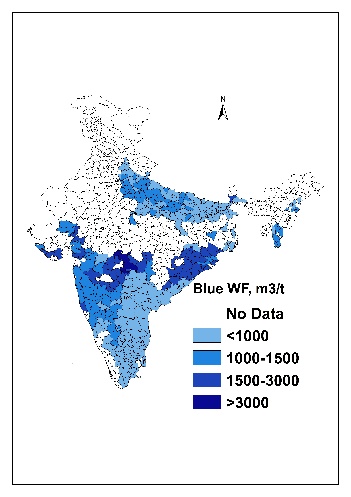** |
| --- | --- | --- | --- |
| **Paddy** | **Wheat** | **Maize (Kharif)** | **Maize (Rabi)** |

Figure S8. Spatial variation in the blue WFs of crops under RCP6.0 2050. These maps were generated using ArcGIS 10.8.1 (<https://www.arcgis.com/index.html>).
